# Wnt5a and Ror2 expression associate with the disease progress of primary thyroid lymphoma

**DOI:** 10.1007/s13277-015-4471-2

**Published:** 2015-11-25

**Authors:** Lei Wang, Dong Yang, Ying-Hou Wang, Xi Li, Hong-Ming Gao, Jun-Yuan Lv, Lei Wang, Shi-Jie Xin

**Affiliations:** 1 Department of Vascular and Thyroid Surgery, the First Affiliated Hospital, China Medical University, Shenyang, China; 2 Department of General Surgery, NO.202 Hospital of PLA, Shenyang, China

**Keywords:** Primary thyroid lymphoma, Wnt5a, Ror2, Immunohistochemistry

## Abstract

Primary thyroid lymphoma (PTL) is a rare malignant thyroid tumor; its pathogenesis is closely related to chronic lymphocytic thyroiditis. The different pathological subtypes and stages of PTL have distinct clinical characteristics and prognosis, but the specific reasons are not clear. Wnt5a is a representative protein of non-canonical Wnt signaling. It plays an important role in many different types of tumors. This study is to explore the changes of Wnt5a and its receptor Ror2 in PTL development process and the clinical significance of their represent. We collected 22 PTL patient tumor specimens and clinical data. We observed the expression of Wnt5a and Ror2 in PTL tumor tissues by immunohistochemistry. Wnt5a was expressed positively in 12 (54.5 %) cases, and Ror2 was expressed positively in 18 (81.8 %) cases. The expression of Wnt5a had a significant difference in different pathological subtypes of PTL (*P* < 0.05). Wnt5a and Ror2 expression were associated with local invasion and clinical stage, respectively (*P* < 0.05), and had no significant correlation with age, gender, and tumor size. Although, no significant difference in overall survival was found between positive and negative groups of Wnt5a (*P* = 0.416) or Ror2 (*P* = 0.256), respectively. We still consider that Wnt5a and Ror2 play a complex and subtle role in the pathogenesis and progression of PTL and may become potential biomarkers and therapeutic targets of PTL.

## Introduction

Primary thyroid lymphoma (PTL) is a rare thyroid malignant tumor, which is defined as a lymphoma involving only the thyroid gland or the thyroid gland and adjacent (regional) neck lymph nodes, without contiguous spread or distant metastases from other areas of involvement at diagnosis [1]. PTL constitutes 1∼5 % of thyroid malignancies, 1∼2 % of extranodal lymphoma [1, 2]. It is five times more common in women and most often presents between 45 and 80 years of age [3, 4]. Dyspnea, dysphagia, and hoarseness are the main symptoms in PTL due to rapid growth and compression of the airways, esophagus, and nerve. The most common histological type of PTL is non-Hodgkin’s lymphoma of B cell origin, diffuse large B cell lymphoma (DLBCL), and mucosa-associated lymphoid tissue (MALT) lymphoma, as the most common pathological subtype, which is accounted for 70 % [3, 5, 6]. However, DLBCL tend to be more aggressive and has a worse prognosis than MALT. In recent years, combination chemotherapy and locoregional radiation are recognized as effective treatment for PTL [7–9]. But overall 5-year survival purview is still between 50 and 60 % [10].

The Wnt signaling pathway is a family of extracellular secretory glycoproteins. These proteins play distinct roles in embryonic development and tumorigenesis, including cell proliferation, migration, polarity, and fate decision [11]. Wnt signaling has two main branches: canonical and non-canonical pathway. Wnt5a was the representative ligand of non-canonical pathway binding to Fzd receptors in conjunction with alternate co-receptors, including orphan tyrosine kinase receptor (Ror2) [12]. Wnt5a is proverbially known as a regulator of cell migration. Its activation has been associated with invasiveness in several tumor types, including breast cancer [13], melanoma [14], pancreatic cancer [15], gastric cancer [16], and osteosarcoma [17]. In most situations, Ror2 is required for Wnt5a-induced cell migration [14, 17, 18].

The reaction of Wnt5a via the Ror2 receptor promotes cell migration and invasion, leading to poor prognosis of these types of tumors. On the contrary, Wnt5a acts as a tumor suppressor in thyroid tumors [19]. Wnt5a is expressed in follicular adenoma (FA), papillary thyroid carcinoma (PTC), and follicular thyroid carcinoma (FTC); no expression has been detected in anaplastic thyroid carcinomas (ATC) or in the normal thyroid [20]. Whether Wnt5a/Ror2 signaling is involved in the development of PTL has not been elucidated. In this study, we investigated the clinical effect of two potential markers Wnt5a and Ror2 expression on PTL.

## Materials and methods

### Patients and tumor samples

All the 22 samples used in this study were collected by the Department of Pathology in First Affiliated Hospital of China Medical University between 2009 and 2013. They were diagnosed with PTL by final pathologic results. We retrospectively reviewed the clinical data of these patients to extract the following information: age, sex, clinical symptoms, tumor size, image finding, histological subtype, treatment procedures, and survival. Tumor invasion was classified into limited in gland and invaded out of the gland. PTL was staged based on the Ann Arbor staging criteria [21]. Stage IE (extranodal) is defined as lymphoma limited to the confines of the thyroid gland, stage IIE denotes spread beyond the thyroid to regional lymph nodes, stage IIIE involves lymph nodes on both sides of the diaphragm, and stage IVE indicates systemic dissemination. All experiments were approved by the hospital’s ethics committee.

### Immunohistochemical staining

Immunohistochemical staining has been performed by using rabbit anti-human Wnt5a antibody (1:100, ab72583, Abcam, USA) and rabbit anti-human Ror2 antibody (1:200, HPA021868, Sigma, Germany). The sections were heated in tissue-drying oven for 1 h at 60 °C, routine deparaffinized, and rehydrated. Antigen retrieval was performed in a microwave for 30 min with citrate buffer, and it was cooled to room temperature and washed with phosphate-buffered saline (PBS). Endogenous peroxidase activity was blocked with endogenous peroxidase blockers for 30 min, then the peroxidase was incubated in 5 % normal goat serum for 30 min. After overnight incubation at 4 °C with the primary antibody, sections were washed with PBS and subsequently incubated with a biotin-conjugated secondary antibody for 1 h at room temperature. The reactions became visible after immersion of diaminobenzidine tetrahydrochloride (DAB).

### Evaluation of immunohistochemical staining results

The cells were regarded as positive for these markers when immunoreactivity was clearly observed in their cell membrane and/or cytoplasm. For each antibody, immunoreactivity (no staining or weak staining less than 10 % of the cells) was scored as negative and other immunoreactivity was scored as positive.

### Statistical analysis

SPSS V.19.0 was used for statistical analysis. The *χ* ^2^ test and Fisher exact test were used for comparison of the immunohistochemistry results. Kaplan-Meier curves were used to estimate overall survival. *P* < 0.05 was considered statistically significant.

## Results

### Patient characteristics

Patients’ mean age was 66.9 years (range 45–89 years); 4 patients were men and 18 were women (ratio 1:4.5). Tumor size was measured by ultrasound. All the patients had thyroglobulin antibody and/or thyromicrosome antibody abnormal increase. All the patients were treated with different ranges of thyroidectomy because most patients had compression symptoms. The excisional specimens were detected by immunohistochemistry to confirmed pathological subtypes; 12 cases were DLBCL, 10 cases were MALT. 9 cases were in stage IE, 13 cases were in stage IIE. Twelve cases were treated with chemotherapy after operation, most R-CHOP (rituxima, cyclophosphamide, doxorubicin, vincristine, prednisolone) or CHOP alone.

### Immunohistochemistry findings

**Fig. 1:**
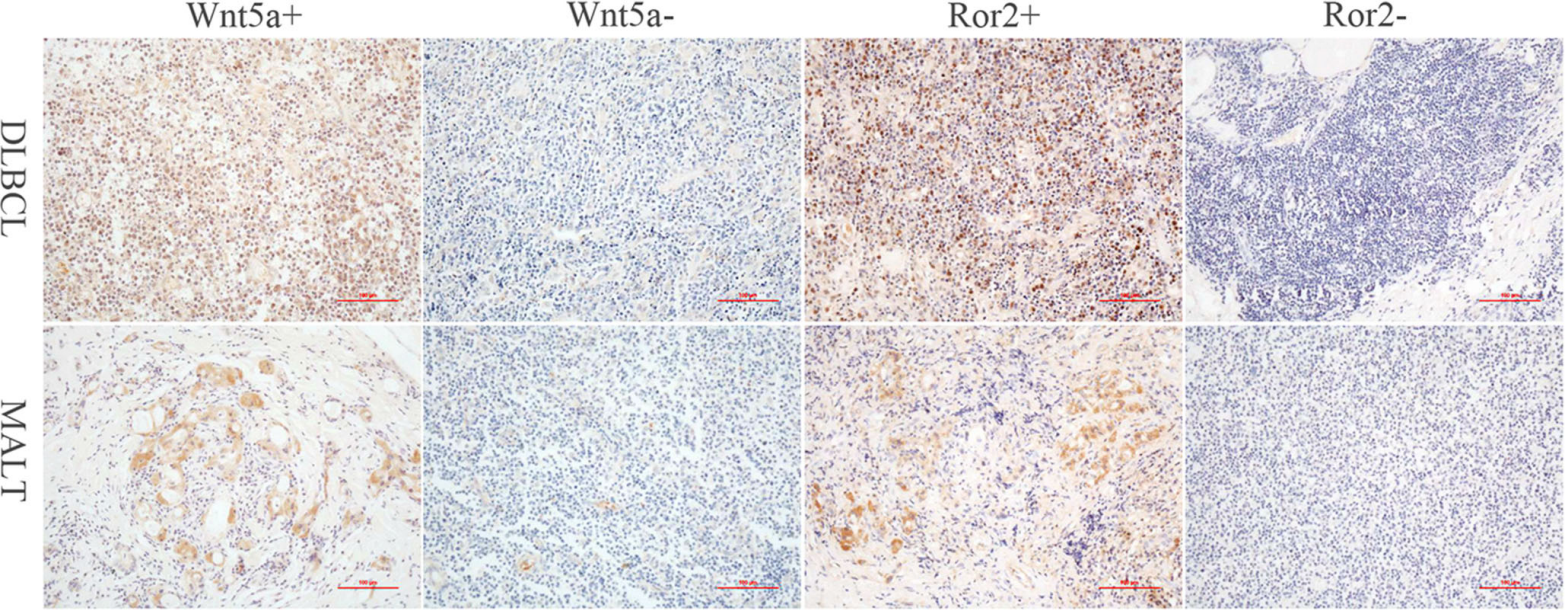
The immunohistochemical staining results of Wnt5a and Ror2 in different pathological subtypes of PTL

Staining of Wnt5a and Ror2 was detected mainly in the cytoplasm (Fig. 1). PTL tumor tissues showed Wnt5a+ and Ror2+ staining in 12 (54.5 %) and 18 (81.8 %) cases, respectively. Among clinicopathological characteristics, sex, age, and tumor size were not significantly associated with the expression of Wnt5a and Ror2 in PTL tissues (*P* > 0.05, Table 1). For distinct pathologic subtype, only four (33.3 %) patients of DLBCL showed Wnt5a positive, meanwhile the MALT patients had 8 (80 %) (*P* < 0.05). We found that Wnt5a was expressed in 25 and 71.4 %, respectively, of limited in gland and invaded out of the gland of PTL goiters (*P* < 0.05). Wnt5a positive expression indicated a significant association with regional invasion. Ror2 was expressed in 55.5 and 100 %, respectively, of clinical stages IE and IIE (*P* < 0.05). Ror2 expression of PTL may induce a disadvantageous stage.

**Table 1:** Association of Wnt5a and Ror2 expression with clinicopathological features

Characteristic	Number of patients	Wnt5a expression		Fisher’s *P* value	Ror2 expression		Fisher’s *P* value
		−	+		−	+	
Age (years)							
<60	7	3	4	0.616	3	4	0.077
≧60	15	7	8		1	14	
Gender							
Female	18	8	10	0.632	4	14	0.418
Male	4	2	2		0	4	
Tumor size (cm)							
<4	6	3	3	0.583	1	5	0.708
≧4	16	7	9		3	13	
Pathologic subtype							
DLBCL	12	8	4	0.038	2	10	0.632
MALT	10	2	8		2	8	
Regional invasion							
Limited in gland	8	6	2	0.048	1	7	0.535
Invaded out gland	14	4	10		3	11	
Clinical stage							
IE	9	5	4	0.853	4	5	0.017
IIE	13	5	8		0	13	

### Correlation of the patient survival with the Wnt5a/Ror2 staining

**Fig. 2:**
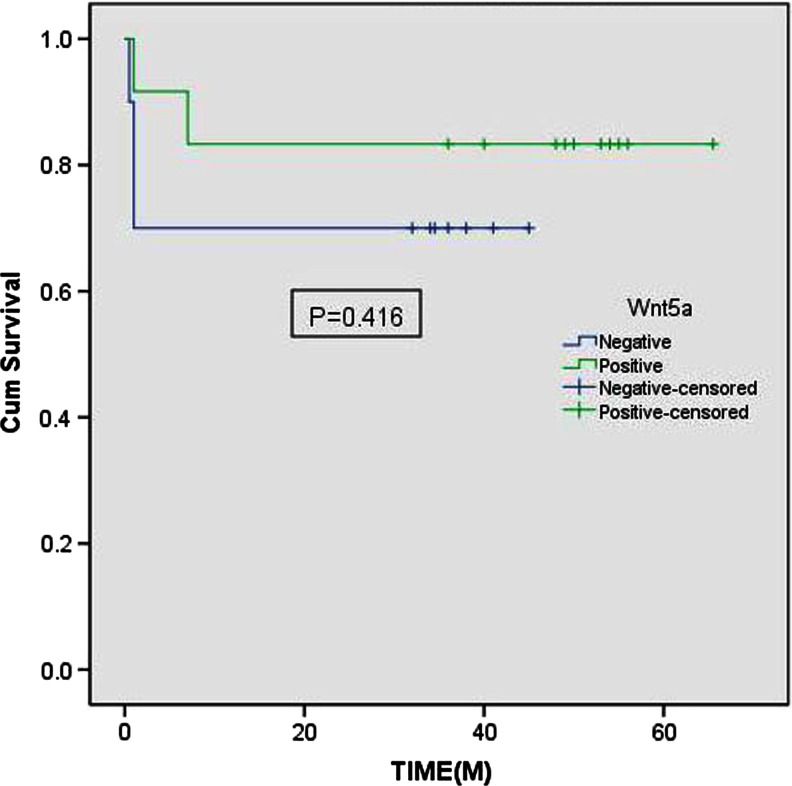
Kaplan-Meier curve: Overall survival for patients with positive (*green*) versus negative (*blue*) Wnt5a expression (cum survival (%), time (month)). There was no significant difference between groups (*P* = 0.416 by log-rank test)

**Fig. 3:**
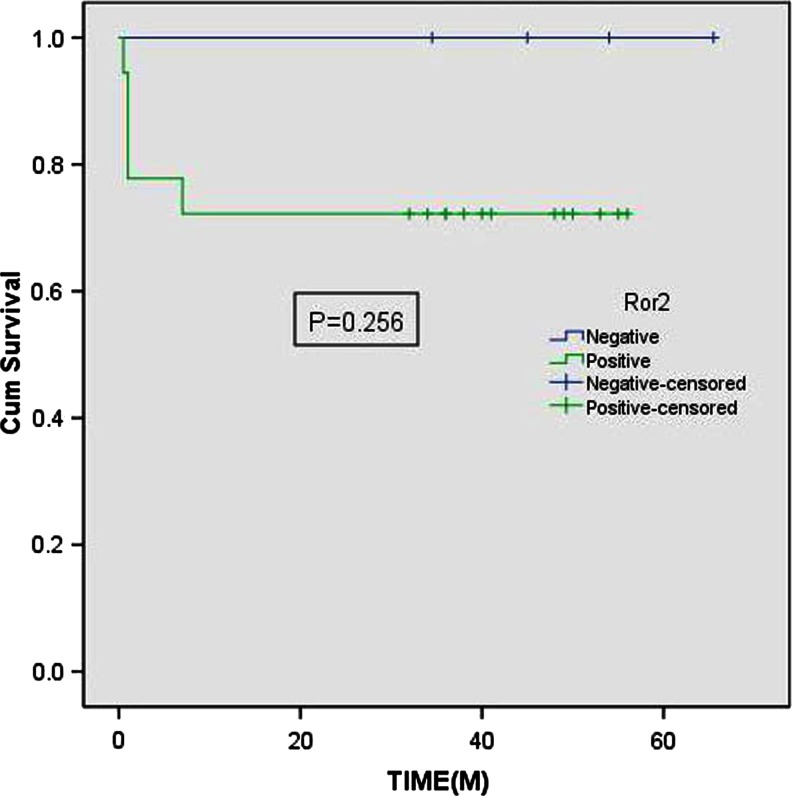
Kaplan-Meier curve: Overall survival for patients with positive (*green*) versus negative (*blue*) Ror2 expression (cum survival (%), time (month)). There was no significant difference between groups (*P* = 0.256 by log-rank test)

All patients were followed up. The longest follow-up time was 66 months. Fourteen cases (64 %) had survived more than 3 years. Five patients (23 %) died in the postoperative half year because of the dissemination of tumor. In the death cases, three patients were with DLBCL and two patients were with MALT. All death cases were not treated with radiotherapy and chemotherapy after operation. Other 17 patients survived. There were no significant difference in overall survival between positive and negative groups of Wnt5a (*P* = 0.416) or Ror2 (*P* = 0.256), respectively. The Kaplan-Meier Curves were shown in Figs. 2 and 3.

## Discussion

Normal thyroid tissue contains no native lymphoid tissue. The pathogenesis of PTL is not entirely clear. The most evidence speculate that chronic autoimmune thyroiditis (Hashimoto’s thyroiditis, HT) has inextricable connection with PTL [22]. Chronic stimulation of antigen may result in lymphocyte atypia hyperplasia and lymphoma. This situation is particularly striking in the occurrence of MALT [3]. Grivennikov et al. [23] research also showed that oncogenesis is associated with chronic inflammation. Mikels et al. [24] observed that Wnt5a played a potential role in the immune regulation. Jungtae et al. [25] further confirmed that Wnt5a signaling might be a target for the regulation of B cell-dependent immunity. Therefore, it is intriguing to speculate that Wnt5a may be involved in the development of PTL.

Wnt5a is one of the most widely studied proteins of Wnt family and is known to play an important role in the development of various organs and diseases. It is involved in the regulation of two non-canonical Wnt pathways, planar cell polarity (PCP)/convergent extension (CE) pathway and Ca^2+^ pathway, and had antagonistic effects on canonical Wnt/β-catenin pathway [26–28]. The interesting ability alternation of Wnt5a signaling between tumor suppressor genes and oncogenes has been exhibited in different cell types and tumors [29–31]. Compared to correlation with poor prognosis in gastric cancer and melanomas [32], Wnt5a was discovered as an antagonist to the canonical Wnt signaling pathway with tumor suppressor activity in differentiated thyroid carcinomas [20]. This mechanism of the negative regulation for the β-catenin pathway by Wnt5a requires Ror2 as a receptor [24]. Meanwhile, Wnt5a could negatively regulate B cell proliferation and suppress hematopoietic malignancies [33]. Our immunohistochemical results found that Wnt5a positive expression in MALT was more than in DLBCL. Previous studies showed that disease-specific survival for patients with DLBCL was significantly shorter compared with MALT lymphoma [4]. According to this point, Wnt5a may play the role of tumor suppressor in PTL.

Ror2 is a single-pass transmembrane receptor with a tyrosine kinase domain and functions as a co-receptor [34]. Wnt5a, Frizzleds (Fz5, Fz6, or Fz7), and Ror2 have been shown to form a ternary complex [35]. This makes that Ror2 may closely participate in the specific activation of Wnt5a signaling. Mikels et al. [24] had approved that Wnt5a required Ror2 as a receptor to negatively regulate the Wnt/β-catenin pathway. Grumolato et al. [36] affirmed that Wnt5a-dependent phosphorylation of Ror2 may be required for activation of the non-canonical pathway which is involved in cell migration. Wnt5a activated through Ror2 regulates the expression of matrix metalloproteases (MMPs) which are the most common target genes related to cancer invasion [17, 37, 38]. In our study, Wnt5a was associated with regional invasion of PTL. Wnt5a expression may play a stimulative role in the tumor invasion to the surrounding tissue. This may be the reason of why most patients combined compression symptoms in advanced stage. Ror2 expression was significantly different between stage IE and IIE. It suggested that Ror2 was involved in the metastasis of PTL. These results are consistent with previous research.

Three-year survival rate of this group of PTL patients was 64 %. The expression of Wnt5a and Ror2 showed no effect on the survival rate in follow-up results. We consider that the results may be affected for three reasons as follows: First, postoperative intervention affected the survival rate. Chemotherapy can be very effective in the treatment of PTL which has been found in previous studies. Five death cases were not treated with chemotherapy after operation. Second, lower incidence of PTL naturally leads to limited sample sizes. We will increase the sample size in subsequent research. Finally, the follow-up time extension was needed. But these did not affect our results obtained in the above experiments.

In summary, Wnt5a and Ror2 had delicate effects in the development of PTL. Our study found that Wnt5a may serve as suppressor in PTL. On the contrary, Wnt5a was associated with tumor regional invasion. Ror2 preferred to express in the disadvantage stage. These suggested that Wnt5a and Ror2 had played a promoting role in tumor metastasis. Our hypothesis is that Wnt5a plays a tumor suppressor role in the early stage of disease. But as the disease progresses, the expression of Wnt5a becomes uncontrollable. Corresponding to the promoting invasion receptor express increase, Wnt5a shows its dark side. We need more studies to confirm this process. But there is no doubt that Wnt5a and Ror2 participate in the development of PTL and may become a breakthrough in its diagnosis and treatment.
